# Association between triglyceride-glucose index and the risk of heart failure hospitalization in older diabetic patients received right ventricular pacing: a retrospective cohort study

**DOI:** 10.1007/s00592-024-02322-0

**Published:** 2024-06-19

**Authors:** Bingqi Fu, Yu Yu, Sijing Cheng, Hao Huang, Tianxin Long, Juweig Yang, Min Gu, Chi Cai, Xuhua Chen, Hongxia Niu, Wei Hua

**Affiliations:** Cardiac Arrhythmia Center, National Center for Cardiovascular Diseases, State Key Laboratory of Cardiovascular Disease, Fuwai Hospital, Chinese Academy of Medical Sciences and Peking Union Medical College, No. 167 Bei Li Shi Rd, Xicheng District, Beijing, 100037 China

**Keywords:** Triglyceride-glucose index, Older, Diabetes, Right ventricular pacing

## Abstract

**Background:**

The prognostic value of triglyceride-glucose (TyG) index is not yet known for older diabetic patients received right ventricular pacing (RVP). We aimed to investigate the association between TyG index and the risk of heart failure hospitalization (HFH) in older diabetic patients received RVP.

**Methods:**

This study was conducted between January 2017 and January 2018 at Fuwai Hospital, Beijing, China, and included older (age ≥ 65 years) diabetic patients that received RVP for the first time. TyG index were obtained before implantation. The primary endpoint was HFH.

**Results:**

A total of 231 patients were divided into three groups according to the tertiles of TyG index: < 8.5 (T1, *N* = 77), 8.5–9.1 (T2, *N* = 77), and > 9.1 (T3, *N* = 77). T3 group had higher rate of HFH (Log-rank = 11.7, *P* = 0.003). Multivariate analyses showed that, TyG index served as an independent predictor for HFH, both as numerical variable (HR = 1.94, 95% CI 1.21–3.11, *P* = 0.006), and as categorical variable (HR = 2.31, 95% CI 1.09–4.89, *P* = 0.03). RCS demonstrated that the risk of HFH was relatively low until TyG index exceeded 8.8, beyond which the risk began to increase rapidly (P-non-linear = 0.006).

**Conclusion:**

Preimplantation TyG index emerges as a robust, independent predictor for HFH in older diabetic patients received RVP, and TyG index > 8.8 might be the optimal cut-off value.

**Supplementary Information:**

The online version contains supplementary material available at 10.1007/s00592-024-02322-0.

## Background

With geriatric population expansion, more attention should be paid on age-related diseases. Aging is a risk factor for metabolic disease. There is a strong association between advanced age and type 2 diabetes mellitus (T2DM), as more than half of diabetic patients were constituted by those aged ≥ 65 years [1, 2]. In addition, T2DM adds additional folds of risk of heart failure (HF), as compared to those without DM [3]. Aging is also a risk factor for cardiac conduction abnormalities [4]. It was reported that in the US, the average age of receiving permanent pacemaker implantation (PPMI) was 73.3–77.5 years in 1993, and steadily increased overtime, reaching 75.4–80.1 years in 2009 [5]. Right ventricular pacing (RVP) is a commonly used pacing strategy, as it is easily accessible and provides stable lead position and low dislodgement rate [6]. However, high percentage of RVP in the long run is associated with abnormal conduction-induced cardiomyopathy and higher rate of heart failure hospitalization (HFH), featured by ventricular asynchrony, hemodynamic change, enlarged ventricles and declined cardiac function [7].

Insulin resistance (IR) is a state of decreased sensitivity and responsiveness to the action of insulin, playing a key role in the pathological mechanism of T2DM [8], and can be evaluated by triglyceride-glucose (TyG) index [9, 10]. Several studies have shown that TyG index is predictive of adverse clinical outcomes. In cardiovascular disease (CVD) patients with T2DM or pre-diabetes, baseline TyG index was associated with cardiovascular death and all-cause mortality [11]. A higher TyG index was also independently associated with incident HF in general population [12]. Nevertheless, whether higher TyG index is associated with worse cardiac function in older diabetic patients receiving RVP is yet unknown. Therefore, this study was design to explore the relationship between TyG index risk of HFH in older diabetic patients receiving RVP.

## Methods

### Study population

**Fig. 1:**
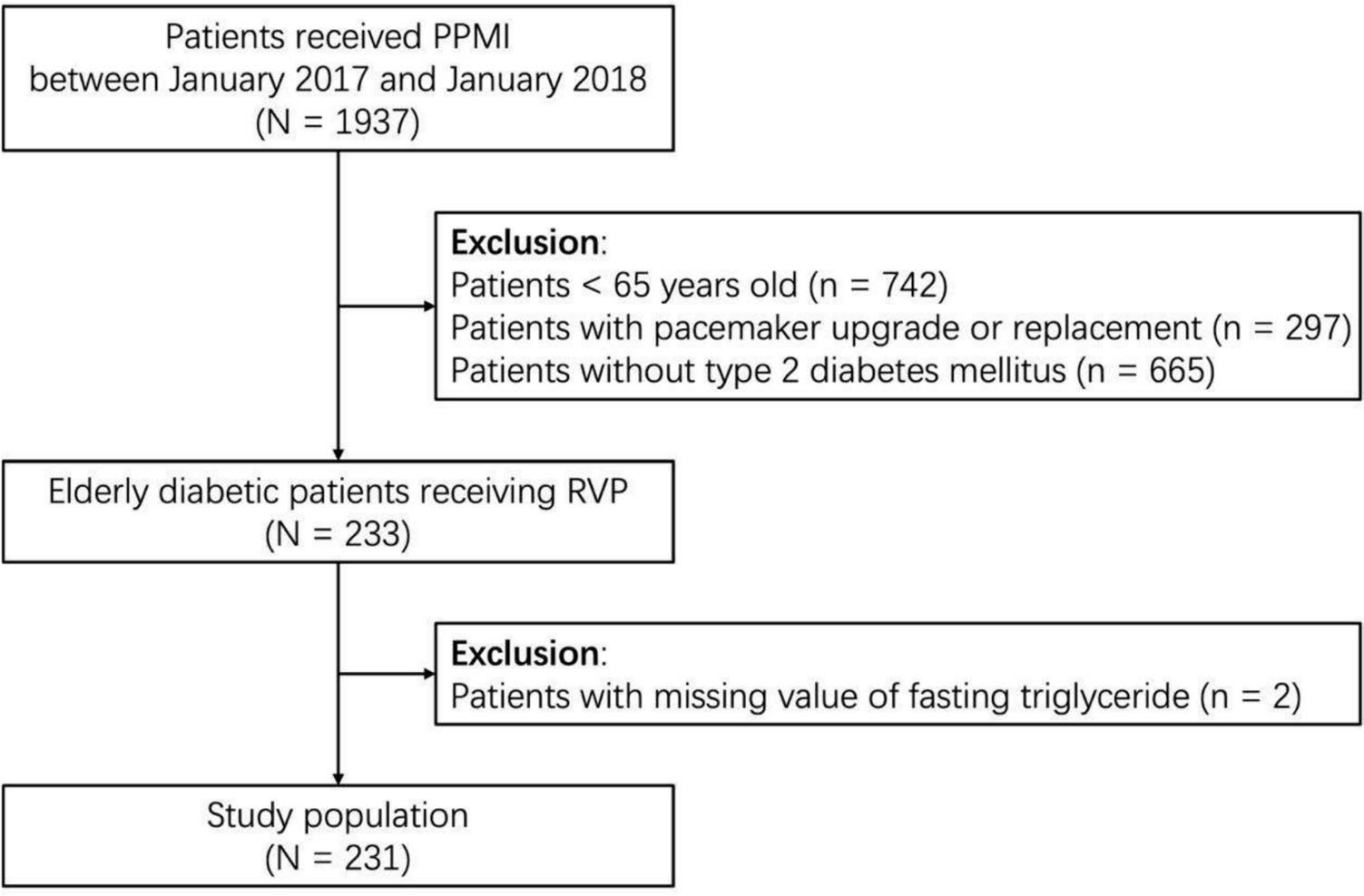
Flowchart of study population. PPMI, permanent pacemaker implantation; RVP, right ventricular pacing

Patients older than 65 years, with the diagnosis of diabetes mellitus, and received RVP for the first time at Fuwai Hospital, Beijing, China, between January 2017 and January 2018 were retrospectively enrolled. There were 1938 patients received PPMI during the study period. After excluding patients aged < 65 years (*N* = 742), with pacemaker upgrade or replacement (*N* = 297), without diagnosis of T2DM (*N* = 665), and with missing values for triglyceride (*N* = 2). Eventually, 231 patients were included in this study (Fig. 1).

The study was approved by the Ethics Committee of the Chinese Academy of Medical Sciences, Fuwai Hospital (No. IRB2012-BG-006). The written informed consent was obtained from all the patients included in this study.

### Data collection and TyG index measurement

Baseline data were extracted from the electronic medical recording system by two independent trained researchers. Any inconsistencies were confirmed by a third researcher.

Demographic information, including age, sex, body mass index, smoke, and alcohol use, past medical history, including sinus node dysfunction, atrioventricular block, hypertension, coronary artery disease, percutaneous coronary intervention (PCI) or coronary artery bypass grafting (CABG), HF, atrial fibrillation (AF), stroke, and chronic kidney disease (CKD), and medical therapy, including angiotensin-converting enzyme inhibitor/angiotensin receptor blocker, β blocker, and statin were obtained upon admission. Physical examination and data of New York Heart Association class, systolic blood pressure, and diastolic blood pressure were recorded later on. The blood samples were collected in a fasting state by trained nurses on the admission day of hospitalization. Laboratory results, including white blood cells, neutrophils, lymphocyte, hemoglobin, platelet, albumin, alanine transaminase, aspartate transaminase, total bilirubin, direct bilirubin, fasting blood glucose (FBG), hemoglobin A1C (HbA1C), estimated glomerular filtration rate (eGFR), triglyceride, total cholesterol (TC), high-density lipoprotein cholesterol (HDL-C), low-density lipoprotein cholesterol (LDL-C), and N-terminal pro-brain natriuretic peptide (NT-proBNP). Echocardiography performed before PPMI, including left atrium diameter, left ventricular ejection fraction (LVEF), left ventricular end-diastolic diameter, and left ventricular mass index, and pacing details, including pacing location and ventricular pacing proportion were also acquired.

TyG index was calculated as: ln[fasting triglycerides (mg/dL) × FBG (mg/dL)/2] before PPMI. Patients were stratified into three groups by the tertiles of TyG index: T1: 6.18–8.53 (*N* = 77), T2: 8.53–9.13 (*N* = 77), and T3: 9.13–11.36 (*N* = 77). The T1 group was set as the reference group.

### Follow-up and study endpoints

The primary outcome of this study was HFH, which is defined any hospitalization necessitated by the new onset or exacerbation of HF symptoms and signs, with significantly elevated levels of NT-proBNP and requiring diuretic therapy. All patients were followed up until January 30th, 2022. The follow-up duration was calculated from the date of receiving RVP, to the data of first incident of HFH, or the data of follow-up deadline. The median follow-up duration was 53 months.

### Statistical analysis

Numerical variables were expressed as mean ± standard deviation if they followed normal distribution, or as median (25th quartile, 75th quartile) if they were skewedly distributed. Categorical variables were expressed as number (percentage). Comparisons of numerical variables among T1, T2, and T3 groups were performed using one-way analysis of variance for those with normal distribution, or using Kruskal–Wallis test for those with skewed distribution, and comparisons of categorical variables were performed using Fisher's exact test or Chi-squared test. Post hoc pairwise comparisons of the variables that showed statistically significant differences across TyG tertiles were performed, using the Wilcoxon rank-sum test, with *P* value adjusted for multiple comparisons via the Bonferroni method. Kaplan–Meier curves were plotted to illustrate the cumulative incidence of HFH across the T1, T2, and T3 group, and Log-rank tests were employed to evaluate the differences among groups. Univariate Cox regression analysis was conducted to identify significant risk factors for HFH, which were incorporated in the multivariate Cox regression analysis. Model 1 was unadjusted. Model 2 with TyG index as a numerical variable and Model 3 with TyG index as a categorical variable, were both adjusted for age, sex, AF, CKD, PCI or CABG, baseline LVEF and LVMI. To evaluate the relationship between TyG index and HFH, Cox proportional hazards regression models with restricted cubic splines (RCS) were conducted. The optimal cut-off value for TyG index on predicting HFH was determined at the point where the HR in the RCS curve exceeded 0. Subgroup analyses were conducted based on age (≥ 75 or < 75 years), sex, AF, CKD, PCI or CABG, and baseline LVEF (≥ 60 or < 60%) and LVMI (≥ 95 or < 95 g/m^2^). For each stratified variable, adjustments were made to control for the potential influence of all remaining variables. The statistical data was analyzed via the R statistical software version 4.3.1. Two-tailed P value < 0.05 was considered statistically significant.

## Results

### Baseline clinical characteristics

The baseline characteristics of study population was shown in Table 1. A total of 231 patients were divided into three groups according to the tertiles of TyG index: < 8.5 (N = 77), 8.5–9.1 (*N* = 77), and > 9.1 (*N* = 77). The median age was 76 years and 48.5% were female patients. The median duration of T2DM was 10 years, and only one patient (0.4%) used sodium-glucose co-transporter-2 (SGLT-2) inhibitors. The mean TyG was 8.8 ± 0.6. Patients with higher TyG index had significantly higher levels of WBC, neutrophil, FBG, HbA1C, TC, and LDL-C, and lower levels of eGFR, and HDL-C, in comparison to those with lower TyG index (all *P* < 0.05). The incidence of HFH was significantly higher in the third tertile (14.3 vs. 13.0 vs. 32.5%, *P* = 0.003).

**Table 1:** Baseline characteristics according to TyG tertiles

	Overall	T1 (< 8.5)	T2 (8.5–9.1)	T3 (> 9.1)	*P* value	P1	P2	P3
*N*, (%)	231	77 (33.3)	77 (33.3)	77 (33.3)				
Age (years)	76 (71,79)	75 (71,80)	76 (72,80)	75 (71,78)	0.440			
Sex, (%)					0.207			
Female	112 (48.5)	31 (40.3)	41 (53.2)	40 (51.9)				
Male	119 (51.5)	46 (59.7)	36 (46.8)	37 (48.1)				
Smoke	77 (33.3)	28 (36.4)	28 (36.4)	21 (27.3)	0.385			
Alcohol	64 (27.7)	24 (31.2)	19 (24.7)	21 (27.3)	0.663			
BMI group, (%)					0.386			
Underweight	9 (3.9)	5 (6.5)	3 (3.9)	1 (1.3)				
Normal	70 (30.3)	27 (35.1)	25 (32.5)	18 (23.4)				
Overweight	110 (47.6)	32 (41.6)	36 (46.8)	42 (54.5)				
Obesity	42 (18.2)	13 (16.9)	13 (16.9)	16 (20.8)				
NYHA class, (%)					0.695			
NYHA class I	217 (93.9)	74 (96.1)	71 (92.2)	72 (93.5)				
NYHA class II-IV	14 (6.1)	3 (3.9)	6 (7.8)	5 (6.5)				
SBP (mmHg)	141.5 ± 19	139.7 ± 19.7	142.6 ± 17.8	142.1 ± 19.4	0.591			
DBP (mmHg)	68.6 ± 10.8	67.6 ± 9.8	70.3 ± 12.1	68.1 ± 10.2	0.257			
*Past medical history, (%)*								
Hypertension	199 (86.1)	64 (83.1)	69 (89.6)	66 (85.7)	0.502			
Coronary artery disease	128 (55.4)	39 (50.6)	45 (58.4)	44 (57.1)	0.581			
PCI or CABG	57 (24.7)	16 (20.8)	19 (24.7)	22 (28.6)	0.533			
Heart failure	50 (21.6)	14 (18.2)	14 (18.2)	22 (28.6)	0.195			
Atrial fibrillation	92 (39.8)	30 (39.0)	30 (39.0)	32 (41.6)	0.930			
Stroke	59 (25.5)	19 (24.7)	22 (28.6)	18 (23.4)	0.744			
Chronic kidney disease	33 (14.3)	10 (13.0)	9 (11.7)	14 (18.2)	0.476			
T2DM duration	10 (7, 15)	10 (5, 14)	10 (7, 16)	10 (10, 17)	0.293			
*Medical therapy, (%)*								
ACEi/ARB	138 (59.7)	45 (58.4)	47 (61.0)	46 (59.7)	0.947			
* β* blocker	129 (55.8)	36 (46.8)	52 (67.5)	41 (53.2)	0.029			
Statin	174 (75.3)	56 (72.7)	61 (79.2)	57 (74.0)	0.613			
*Laboratory results*								
White blood cell, × 10^9^/L	6.5 (5.5,8.0)	6.2 (5.0,7.1)	6.2 (5.4,8.0)	6.9 (6.0,8.1)	**0.009**	0.745	**0.007**	0.184
Neutrophil, × 10^9^/L	4.0 (3.2,5.1)	3.5 (2.9,4.7)	4.0(3.4,5.0)	4.4 (3.2,5.8)	**0.021**	0.099	**0.031**	1.000
Lymphocyte, × 10^9^/L	1.8 ± 0.6	1.7 ± 0.5	1.7 ± 0.7	1.8 ± 0.7	0.233			
Hemoglobin, g/L	130.4 ± 18.4	127.5 ± 18.9	131.3 ± 17.3	132.2 ± 18.8	0.247			
Platelet, × 10^9^/L	197 (154,228)	180 (139,209)	206 (155,240)	202 (164,245)	0.028^*^	0.079	0.051	1.000
Albumin, g/L	41.8 (38.6,45.6)	41.9 (37.2,45.5)	42 (38.2,46.0)	41.5 (38.9,45.5)	0.599			
ALT, U/L	17 (13,25)	18 (13,23)	17 (12,27)	17 (13,25)	0.697			
AST, U/L	20 (17,25)	20 (17,24)	20 (16,25)	20 (16,25)	0.869			
Total bilirubin, µmol/L	13.7 (10.7,18.1)	13.6 (11.2,17.4)	13.1 (10.7,18.4)	14.4 (10.7,18.1)	0.815			
Direct bilirubin, µmol/L	2.6 (1.9,3.3)	2.6 (2.1,3.5)	2.3 (1.8,3.2)	2.5 (1.8,3.3)	0.185			
FBG, mmol/L	6.7 (5.6,8.3)	5.5 (4.9,6.6)	6.7 (5.9,7.7)	8.5 (7.2,10.3)	** < 0.001**	**0.003**	** < 0.001**	**0.003**
HbA1C, %	6.9 (6.3,7.7)	6.6 (6.1,7)	7.0 (6.3,7.7)	7.5 (6.8,8.2)	** < 0.001**	**0.017**	** < 0.001**	**0.013**
eGFR, ml/min/1.73m^2^	68.9 (58.7,77.0)	71.9 (63.4,79.0)	68.9 (61.9,78.1)	65.2 (52.3,74.7)	**0.030**	0.900	**0.040**	0.200
TG, mmol/L	1.3 (0.9,1.6)	0.8 (0.7,0.9)	1.3 (1.1,1.5)	1.8 (1.5,2.2)	** < 0.001**	** < 0.001**	** < 0.001**	** < 0.001**
TC, mmol/L	3.8 (3.3,4.4)	3.6 (3.0,4.1)	3.8 (3.3,4.4)	3.9 (3.4,4.5)	0.025			
HDL-C, mmol/L	1.1 (0.9,1.4)	1.3 (1,1.5)	1.1 (0.9,1.4)	1.0 (0.8,1.2)	** < 0.001**	0.082	**0.029**	0.068
LDL-C, mmol/L	2.1 (1.7,2.6)	1.9 (1.5,2.5)	2.2 (1.9,2.8)	2.2 (1.8,2.8)	**0.010**	**0.015**	0.053	1.000
NT-proBNP, pg/mL	357.7 (158.6,954.6)	442 (163.1,1022.0)	280.2 (158.8,828.2)	371.2 (155.6,1219.0)	0.285			
*Echocardiographic features*								
LAD, mm	40 (36,43)	40 (37,43)	40 (38,43)	39 (35,44)	0.592			
LVEF, %	63 (60,65)	63 (60,65)	63 (60,65)	62 (60,65)	0.437			
LVEDD, mm	48 (45,52)	48 (46,51)	48 (45,52)	48 (45,51)	0.838			
LVMI, g/m^2^	92.2 (79.6,109.4)	92.2 (78.1,107.2)	92.8 (81.6,107.7)	91.9 (82.7,113.6)	0.854			
*Pacing indications, (%)*								
SND	154 (66.7)	50 (64.9)	58 (75.3)	46 (59.7)	0.113			
AVB	78 (33.8)	29 (37.7)	22 (28.6)	27 (35.1)	0.470			
AF with slow HR	20 (8.7)	5 (6.5)	6 (7.8)	9 (11.7)	0.491			
*Pacing details, (%)*								
Right ventricular septum	81 (35.1)	29 (37.7)	28 (36.4)	24 (31.2)	0.671			
Right ventricular apex	150 (64.9)	48 (62.3)	49 (63.6)	53 (68.8)	0.671			
VP ≥ 40%	78 (33.8)	28 (36.4)	21 (27.3)	29 (37.7)	0.332			
*HFH, (%)*	46 (19.9)	11 (14.3)	10 (13.0)	25 (32.5)	**0.003**			

The *p*-value represents the comparison among the three tertiles. P1 is for the comparison between T1 and T2, P2 is for the comparison between T1 and T3, and P3 is for the comparison between T2 and T3

TyG index, triglyceride-glucose index; BMI, body mass index; NYHA, New York Heart Association; SBP, systolic blood pressure; DBP, diastolic blood pressure; T2DM, type 2 diabetes mellitus; PCI, percutaneous coronary intervention; CABG, coronary artery bypass grafting; ACEi/ARB, angiotensin-converting enzyme inhibitor/angiotensin receptor blocker; ALT, alanine transaminase; AST, aspartate transaminase; FBG, fasting blood glucose; HbA1C, hemoglobin A1C; eGFR, estimated glomerular filtration rate; TG, triglyceride; TC, total cholesterol; HDL-C, high-density lipoprotein cholesterol; LDL-C, low-density lipoprotein cholesterol; NT-proBNP, N-terminal pro-brain natriuretic peptide; LAD, left atrium diameter; LVEF, left ventricular ejection fraction; LVEDD, left ventricular end-diastolic diameter; LVMI, left ventricular mass index; SND, sinus node dysfunction; AVB, atrioventricular block; AF, atrial fibrillation; HR, heart rate; VP, ventricular pacing; HFH, heart failure hospitalization

^*^Significant overall *p*-value with non-significant post hoc results might due to small sample size

Bold text is to highlight statistically significant P values

### Relationships of TyG index with heart failure hospitalization

**Fig. 2:**
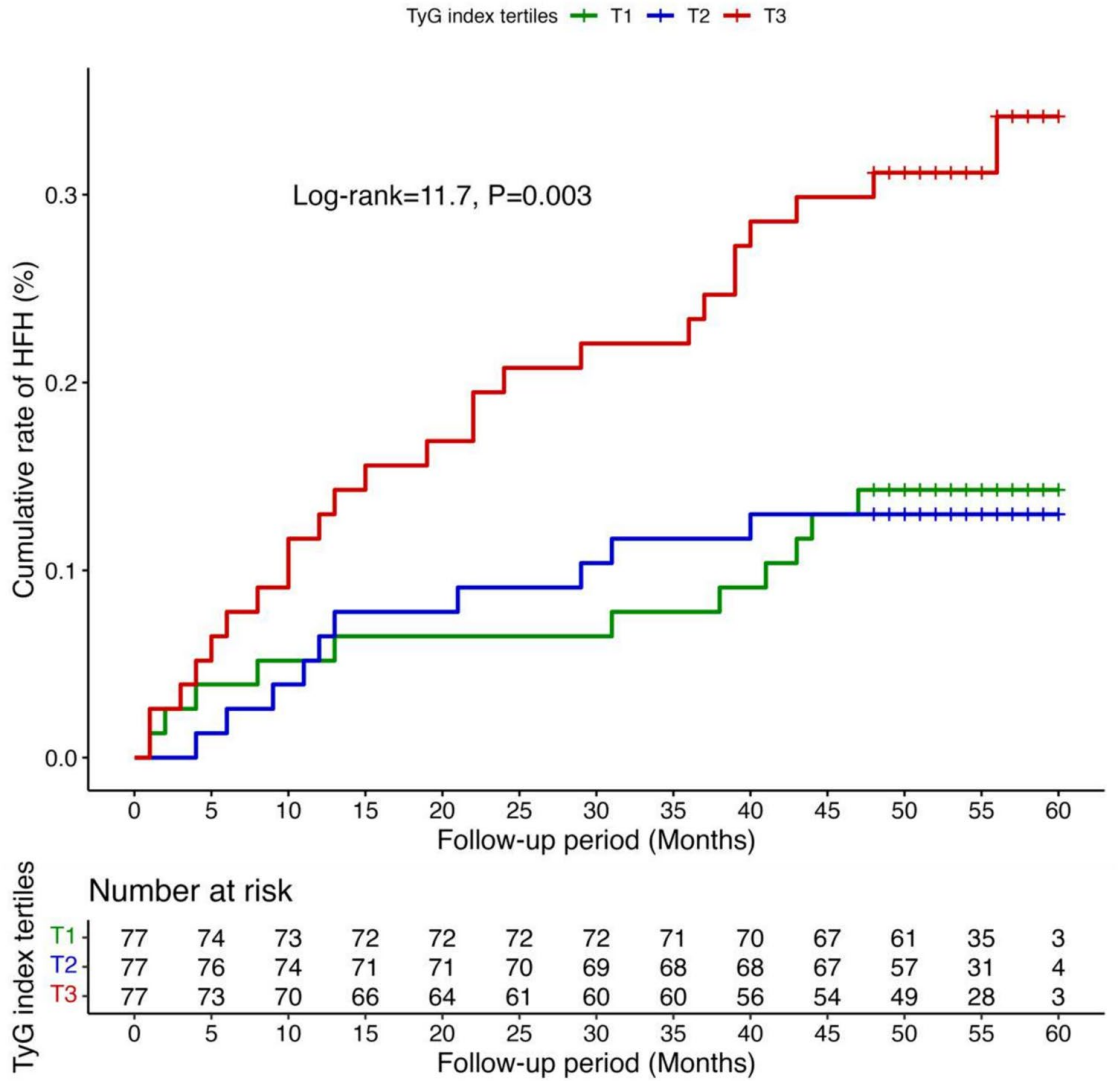
The cumulative rate of HFH according to TyG tertiles. HFH, heart failure hospitalization; TyG index, triglyceride-glucose index

Kaplan–Meier analysis showed that TyG index > 9.1 had higher cumulative rate of HFH during follow-up period (Log-rank = 11.7, P = 0.003; Fig. 2). Univariate Cox regression analysis revealed that, as a numerical variable, TyG index was significantly associated with an elevated risk of HFH (HR = 2.12, 95% CI 1.33–3.37, *P* = 0.002; Table 2). As a categorical variable, TyG index in T3 group was significantly correlated with increased risk of HFH (HR = 2.54, 95% CI 1.25–5.16, *P* = 0.010; Table 2).

**Table 2:** Cox regression analyses for the association between TyG index and HFH

	Model 1^1^	Model 2^2#^			Model 3^3#^		
	HR (95% CI)	HR	95% CI	*P* value	HR	95% CI	*P* value
Age	1.06 (1.01–1.11)	1.04	1.00–1.09	0.074	**1.05**	1.00–1.10	**0.040**
Male	1.15 (0.64–2.05	0.79	0.42–1.50	0.481	0.85	0.45–1.61	0.623
AF	1.93 (1.08–3.44)	1.36	0.72–2.56	0.337	1.40	0.74–2.62	0.300
CKD	4.13 (2.25–7.59)	**2.76**	1.43–5.35	**0.003**	**2.80**	1.44–5.44	**0.002**
PCI or CABG	2.12 (1.17–3.84)	1.85	1.00–3.45	0.052	**1.93**	1.03–3.61	**0.041**
Baseline LVEF	0.91 (0.87–0.95)	**0.93**	0.88–0.99	**0.019**	**0.92**	0.87–0.97	**0.004**
Baseline LVMI	1.02 (1.01–1.03)	1.01	0.99–1.02	0.245	1.01	0.99–1.02	0.413
TyG index	2.12 (1.33–3.37)	**1.94**	1.21–3.11	**0.006**	–	–	–
TyG index T1	Reference	–	–	–	Reference	–	–
TyG index T2	0.92 (0.39–2.17)	–	–	–	0.69	0.29–1.67	0.409
TyG index T3	2.54 (1.25–5.16)	–	–	–	**2.31**	1.09–4.89	**0.028**

HFH, heart failure rehospitalization; HR, hazard ratio; CI, confidential interval; AF, atrial fibrillation; CKD, chronic kidney disease; PCI, percutaneous coronary intervention; CABG, coronary artery bypass grafting; LVEF, left ventricular ejection fraction; LVMI, left ventricular mass index; TyG index, triglyceride-glucose index

^1^Model 1: unadjusted

^2^Model 2: TyG index adjusted as a numerical variable

^3^Model 3: TyG index adjusted as a categorical variable

^#^Both model 2 and 3 were adjusted for age, sex, AF, CKD, PCI or CABG, baseline LVEF and LVMI

Bold text is to highlight statistically significant P values

Multivariate Cox regression models were built to evaluate the independent association between TyG index and HFH. In model 2, where TyG index was adjusted as a numerical variable, TyG index served as an independent predictor for HFH, with each unit increase being associated with a 94% elevation in the risk of HFH (HR = 1.94, 95% CI 1.21–3.11, *P* = 0.006; Table 2). In model 3, where TyG was adjusted as a categorical variable, TyG index in T3 group was an independent predictor for HFH (HR = 2.31, 95% CI 1.09–4.89, *P* = 0.028; Table 2).

### The detection of non-linear relationship

**Fig. 3:**
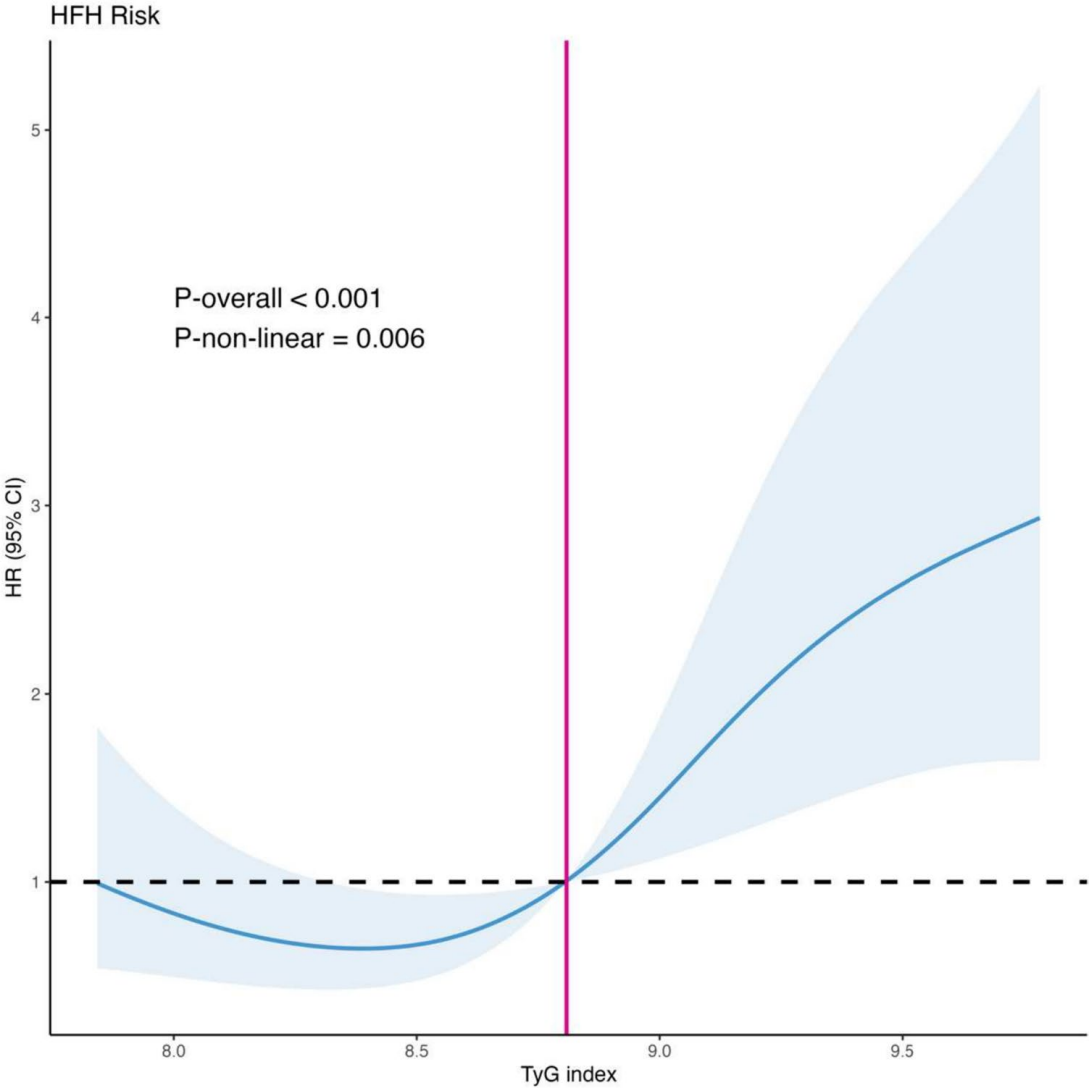
Restricted cubic splines regression analysis of TyG index with HFH risk. HFH, heart failure hospitalization; TyG index, triglyceride-glucose index

RCS curves were plotted to assess the non-linear relationship between TyG index and the risk of HFH. Overall, the risk of HFH was relatively low, until TyG index exceeded 8.8, beyond which the risk began to increase rapidly (*P*-non-linear = 0.006; Fig. 3). Similar non-linear relationships were also observed in male patients (P-non-linear = 0.048; Figure S1), patients with AF (*P*-non-linear = 0.047; Figure S2), and patients without CKD (*P*-non-linear = 0.046; Figure S3).

### Subgroup analysis

To further evaluate the association between covariates and HFH, patients were stratified based on age, sex, AF, CKD, PCI or CABG, baseline LVEF and LVMI. The results of multivariate Cox regression analyses and forest plots were shown in Figure S4. In patients with age ≥ 75 years (HR = 1.79, 95% CI 1.002–3.18, *P* = 0.049), female gender (HR = 4.07, 95% CI 1.62–10.22, *P* = 0.003), AF (HR = 3.07, 95% CI 1.58–5.98, *P* = 0.001), baseline LVEF < 60% (HR = 3.07, 95% CI 1.004–9.42, *P* = 0.049), and patients without PCI or CABG (HR = 2.91, 95% CI 1.57–5.38, *P* = 0.001), TyG index were significantly associated with HFH. Specifically, there was significant interaction of TyG index with AF (*P* interaction = 0.030) and PCI or CABG (*P* interaction = 0.035).

## Discussion

This study investigated the association between TyG index and HFH in older diabetic patients receiving RVP. Our results showed that, TyG index was positively correlated with increased risk of HFH, both as numerical and categorial variable. After adjusting possible confounding factors, TyG exhibited as an independent predictor for HFH. In addition, RCS curves revealed a non-linear relationship between TyG index and the risk of HFH; specifically, the risk of HFH was relatively low, until TyG index exceeded 8.8, beyond which the risk began to increase rapidly. To the best of our knowledge, the study was the first to demonstrate the potential usefulness of TyG index, a simple indicator of IR, on discriminating high risk for HFH in older diabetic patients receiving RVP.

IR is a key pathological mechanism in T2DM and a risk factor for CVD [10, 13]. Various methods exist for assessing IR. The hyperinsulinemic euglycemic clamp (HEC) technique is considered the most accurate, yet its complexity limits its use to small-scale research rather than large population studies. An alternative, the homeostasis model assessment of IR (HOMA-IR), correlates well with HEC results. However, the requirement for fasting insulin levels renders it less practical for widespread clinical use in community settings. Consequently, the TyG index has been developed. This index substitutes the measurement of insulin with triglycerides, facilitating a quicker evaluation of IR. Importantly, it retains a consistent correlation with HEC and HOMA-IR values, offering a more feasible approach for broad clinical application [14, 15].

TyG index has demonstrated significant clinical relevance in patients with T2DM. Zhang et al. [11] involved patients with T2DM and CVD, and revealed a positive correlation between TyG index and future CVD death and all-cause mortality. Wang et al. [16] recruited patients with T2DM and acute coronary syndrome, and found that a higher TyG index was associated with higher risk of major adverse cardiovascular events (MACEs), defined as all-cause death, non-fatal myocardial infarction, and non-fatal stroke. In patients with T2DM that underwent PCI, Chen et al. [17] was able to identify elevated TyG index as a feasible predictor for recurrent revascularization. In patients with T2DM and acute ischemic stroke, Liu et al. [18] showed that increased TyG index was strongly related to recurrency of ischemic stroke and all-cause death. Thus, it is essential to pay attention to TyG index in diabetic patients.

Additionally, TyG index has previously been shown to be effective in predicting health concerns such as critical delirium, frailty, and arterial stiffness in the elderly [19–21]. Despite these findings, research specifically targeting older diabetic patients remains scarce. Zhao et al. [22] identified a correlation between a high TyG index and increased mortality in elderly diabetic patients. Similarly, Huang et al. [23] focused on elderly female patients with diabetic foot ulcers and discovered a strong association between the TyG index and all-cause mortality, further underscoring TyG index’s potential as a valuable prognostic tool in older diabetic patients.

In regards to cardiac-related endpoints, high TyG index exhibited as a feasible predictor for heart failure development and exacerbation. In general population, TyG index has been identified as an independent risk factor for incident HF, as demonstrated in studies by Li et al. [12] and Xu et al. [24]. This correlation was also significant in patients who have undergone PCI and subsequently developed secondary mitral regurgitation. In Huang et al.’s study, an elevated preprocedural TyG index may signal an increased risk of worsening HF [25]. Adding to this, Zheng et al. [26] highlighted that prolonged exposure to high TyG index levels was associated with an escalated risk of HF. In the context of patients with T2DM, studies by Wang et al. [27] and Chen et al. [28] showed a significant association between the TyG index and subclinical cardiac function decline. Our study focused on older diabetic patients undergoing RVP, a group inherently more susceptible to declining cardiac function over time. In line with previous findings, we observed a positive correlation between TyG index and an increased risk for HFH in this specific cohort.

It is increasingly recognized that IR is closely associated with the development of cardiomyopathy. Primarily, IR leads to an excessive breakdown of triglycerides and release of free fatty acids from adipose tissue. These fatty acids circulate in the bloodstream, accumulate in cardiomyocytes, cause mitochondrial dysfunction and endoplasmic reticulum stress, and subsequently trigger the release of pro-inflammatory and fibrogenic mediators and activate fibroblasts, contributing to cardiac fibrosis [29–32]. Furthermore, IR plays a role in activating the renin–angiotensin–aldosterone system, producing oxidative stress, altering mitochondrial function, exacerbating cardiac diastolic dysfunction and cardiac remodeling [33, 34]. Lastly, IR adversely affects calcium handling, a critical factor in modulating myocardial contractility and relaxation. The impairment can also manifest as decreased cardiac diastolic function [35–37].

We also involved plotting RCS curves, which unveiled a non-linear relationship between the TyG index and the risk of HFH in the older diabetic population. Intriguingly, we observed that the risk of HFH remained relatively low until the TyG index surpasses 8.8. This non-linear relationship and the identified threshold align with findings from previous studies focusing on older population [19, 21, 22]. Some research even suggested a U-shaped relationship, indicating that both excessively low and high TyG index values were linked to poorer prognoses [11]. Our study did not detect a U-shaped association between TyG index and HFH, which may be due to several reasons. For one thing, the primary endpoint was different. Our results were similar to the majority of studies that focused on using TyG index to predict HF, where a U-shaped association was also not observed [24–26]. This suggests that the relationship between the TyG index and HF risk may follow a different pattern compared to cardiovascular or metabolic endpoints. For another, our study population all received RVP. The mechanisms of developing HF in our population may include pathological process like prolonged ventricular pacing, ventricular contraction asynchrony, and gradual cardiac function loss [38], which added complexity beyond what was typically seen in patients with advanced age, T2DM or CVD. Further research with larger sample size is required to determine whether a low TyG index is associated with adverse outcomes in older diabetic patients receiving RVP.

This study is subject to several limitations. Firstly, it is a retrospective cohort study with a relatively small sample size lacking variable such as SGLT-2 inhibitors use. Our findings, including the identified optimal threshold and the non-linear relationship between TyG index and HFH risk, should be verified in randomized-controlled trials (RCTs) involving larger populations. Secondly, while efforts were made to adjust for potential covariates, there might be residual confounding factors influencing the outcome. Thirdly, the underlying mechanisms driving the association between TyG index and HFH risk in the older diabetic patietns receiving RVP remain unclear. Experimental studies are essential to provide deeper insights into the biological processes and causal pathways involved, thereby enhancing our understanding of the role of TyG index in predicting compromised cardiac function.

## Conclusion

Preimplantation TyG index was positively correlated with an increased risk of HFH in older diabetic patients receiving RVP, serving as an independent predictor even after adjusting for potential confounders. This correlation was observed both when TyG index was treated as a numerical and as a categorical variable. Notably, RCS curves identified a non-linear relationship between TyG index and HFH risk. The risk remained relatively low until the TyG index surpassed the threshold of 8.8, beyond which the risk escalated significantly. Therefore, our study underscored the potential utility of preimplantation TyG index in identifying a higher risk for HFH among older diabetic patients receiving RVP, allowing closer follow-up and timely management.

## Supplementary Information

Below is the link to the electronic supplementary material.

Supplementary file1 (DOCX 14330 KB)

## Data Availability

The raw data supporting the conclusions of this article will be made available by the authors, without undue reservation.

## Abbreviations

AF: Atrial fibrillation
CABG: Coronary artery bypass grafting
CKD: Chronic kidney disease
CVD: Cardiovascular disease
eGFR: Estimated glomerular filtration rate
FBG: Fasting blood glucose
HbA1C: Hemoglobin A1C
HDL-C: High-density lipoprotein cholesterol
HEC: Hyperinsulinemic euglycemic clamp
HF: Heart failure
HFH: Heart failure hospitalization
HOMA-IR: Homeostasis model assessment of insulin resistance
IR: Insulin resistance
LDL-C: Low-density lipoprotein cholesterol
LVEF: Left ventricular ejection fraction
NT-proBNP: N-terminal pro-brain natriuretic peptide
PCI: Percutaneous coronary intervention
PPMI: Permanent pacemaker implantation
RCS: Restricted cubic splines
RVP: Right ventricular pacing
T2DM: Type 2 diabetes mellitus
TC: Total cholesterol
TyG index: Triglyceride-glucose index

## References

[1] Amorim JA, Coppotelli G, Rolo AP, Palmeira CM, Ross JM, Sinclair DA (2022) Mitochondrial and metabolic dysfunction in ageing and age-related diseases. Nat Rev Endocrinol 18(4):243–258. 10.1038/s41574-021-00626-7 35145250 10.1038/s41574-021-00626-7 PMC9059418

[2] Bellary S, Kyrou I, Brown JE, Bailey CJ (2021) Type 2 diabetes mellitus in older adults: clinical considerations and management. Nat Rev Endocrinol 17(9):534–548. 10.1038/s41574-021-00512-2 34172940 10.1038/s41574-021-00512-2

[3] Tan Y, Zhang Z, Zheng C, Wintergerst KA, Keller BB, Cai L (2020) Mechanisms of diabetic cardiomyopathy and potential therapeutic strategies: preclinical and clinical evidence. Nat Rev Cardiol 17(9):585–607. 10.1038/s41569-020-0339-2 32080423 10.1038/s41569-020-0339-2 PMC7849055

[4] Kusumoto FM, Schoenfeld MH, Barrett C, Edgerton JR, Ellenbogen KA, Gold MR et al (2019) 2018 ACC/AHA/HRS guideline on the evaluation and management of patients with bradycardia and cardiac conduction delay: a report of the American college of cardiology/American heart association task force on clinical practice guidelines and the heart rhythm society. J Am Coll Cardiol 74(7):e51–e156. 10.1016/j.jacc.2018.10.044 30412709 10.1016/j.jacc.2018.10.044

[5] Greenspon AJ, Patel JD, Lau E, Ochoa JA, Frisch DR, Ho RT et al (2012) Trends in permanent pacemaker implantation in the United States from 1993 to 2009: increasing complexity of patients and procedures. J Am Coll Cardiol 60(16):1540–1545. 10.1016/j.jacc.2012.07.017 22999727 10.1016/j.jacc.2012.07.017

[6] Liu L, Tang J, Peng H, Wu S, Lin C, Chen D et al (2015) A long-term, prospective, cohort study on the performance of right ventricular pacing leads: comparison of active-fixation with passive-fixation leads. Sci Rep 5:7662. 10.1038/srep07662 25563218 10.1038/srep07662 PMC4288218

[7] Huizar JF, Kaszala K, Tan A, Koneru J, Mankad P, Kron J et al (2023) Abnormal conduction-induced cardiomyopathy: JACC review topic of the week. J Am Coll Cardiol 81(12):1192–1200. 10.1016/j.jacc.2023.01.040 36948737 10.1016/j.jacc.2023.01.040 PMC10715964

[8] Santoro A, Kahn BB (2023) Adipocyte regulation of insulin sensitivity and the risk of type 2 diabetes. N Engl J Med 388(22):2071–2085. 10.1056/NEJMra2216691 37256977 10.1056/NEJMra2216691

[9] Brito ADM, Hermsdorff HHM, Filgueiras MS, Suhett LG, Vieira-Ribeiro SA, Franceschini S et al (2021) Predictive capacity of triglyceride-glucose (TyG) index for insulin resistance and cardiometabolic risk in children and adolescents: a systematic review. Crit Rev Food Sci Nutr 61(16):2783–2792. 10.1080/10408398.2020.1788501 32744083 10.1080/10408398.2020.1788501

[10] Tao LC, Xu JN, Wang TT, Hua F, Li JJ (2022) Triglyceride-glucose index as a marker in cardiovascular diseases: landscape and limitations. Cardiovasc Diabetol 21(1):68. 10.1186/s12933-022-01511-x 35524263 10.1186/s12933-022-01511-x PMC9078015

[11] Zhang Q, Xiao S, Jiao X, Shen Y (2023) The triglyceride-glucose index is a predictor for cardiovascular and all-cause mortality in CVD patients with diabetes or pre-diabetes: evidence from NHANES 2001–2018. Cardiovasc Diabetol 22(1):279. 10.1186/s12933-023-02030-z 37848879 10.1186/s12933-023-02030-z PMC10583314

[12] Li X, Chan JSK, Guan B, Peng S, Wu X, Lu X et al (2022) Triglyceride-glucose index and the risk of heart failure: evidence from two large cohorts and a mendelian randomization analysis. Cardiovasc Diabetol 21(1):229. 10.1186/s12933-022-01658-7 36329456 10.1186/s12933-022-01658-7 PMC9635212

[13] Ding X, Wang X, Wu J, Zhang M, Cui M (2021) Triglyceride-glucose index and the incidence of atherosclerotic cardiovascular diseases: a meta-analysis of cohort studies. Cardiovasc Diabetol 20(1):76. 10.1186/s12933-021-01268-9 33812373 10.1186/s12933-021-01268-9 PMC8019501

[14] Tahapary DL, Pratisthita LB, Fitri NA, Marcella C, Wafa S, Kurniawan F et al (2022) Challenges in the diagnosis of insulin resistance: focusing on the role of HOMA-IR and Tryglyceride/glucose index. Diabetes Metab Syndr 16(8):102581. 10.1016/j.dsx.2022.102581 35939943 10.1016/j.dsx.2022.102581

[15] Anoop S, Jebasingh FK, Rebekah G, Kurian ME, Mohan VR, Finney G et al (2020) The triglyceride/glucose ratio is a reliable index of fasting insulin resistance: observations from hyperinsulinaemic-euglycaemic clamp studies in young, normoglycaemic males from southern India. Diabetes Metab Syndr 14(6):1719–1723. 10.1016/j.dsx.2020.08.017 32916555 10.1016/j.dsx.2020.08.017

[16] Wang L, Cong HL, Zhang JX, Hu YC, Wei A, Zhang YY et al (2020) Triglyceride-glucose index predicts adverse cardiovascular events in patients with diabetes and acute coronary syndrome. Cardiovasc Diabetol 19(1):80. 10.1186/s12933-020-01054-z 32534586 10.1186/s12933-020-01054-z PMC7293784

[17] Chen Q, Xiong S, Zhang Z, Yu X, Chen Y, Ye T et al (2023) Triglyceride-glucose index is associated with recurrent revascularization in patients with type 2 diabetes mellitus after percutaneous coronary intervention. Cardiovasc Diabetol 22(1):284. 10.1186/s12933-023-02011-2 37865753 10.1186/s12933-023-02011-2 PMC10590524

[18] Liu D, Yang K, Gu H, Li Z, Wang Y, Wang Y (2022) Predictive effect of triglyceride-glucose index on clinical events in patients with acute ischemic stroke and type 2 diabetes mellitus. Cardiovasc Diabetol 21(1):280. 10.1186/s12933-022-01704-4 36510223 10.1186/s12933-022-01704-4 PMC9743618

[19] Yuan Y, Chen S, Lin C, Huang X, Lin S, Huang F et al (2023) Association of triglyceride-glucose index trajectory and frailty in urban older residents: evidence from the 10-year follow-up in a cohort study. Cardiovasc Diabetol 22(1):264. 10.1186/s12933-023-02002-3 37775740 10.1186/s12933-023-02002-3 PMC10542691

[20] Su Y, Wang S, Sun J, Zhang Y, Ma S, Li M et al (2021) Triglyceride glucose index associated with arterial stiffness in chinese community-dwelling elderly. Front Cardiovasc Med 8:737899. 10.3389/fcvm.2021.737899 34589530 10.3389/fcvm.2021.737899 PMC8473610

[21] Huang X, Cheng H, Yuan S, Ling Y, Tan S, Tang Y et al (2023) Triglyceride-glucose index as a valuable predictor for aged 65-years and above in critical delirium patients: evidence from a multi-center study. BMC Geriatr 23(1):701. 10.1186/s12877-023-04420-0 37904099 10.1186/s12877-023-04420-0 PMC10617052

[22] Zhao M, Xiao M, Tan Q, Lu F (2023) Triglyceride glucose index as a predictor of mortality in middle-aged and elderly patients with type 2 diabetes in the US. Sci Rep 13(1):16478. 10.1038/s41598-023-43512-0 37777574 10.1038/s41598-023-43512-0 PMC10542790

[23] Huang X, Han J, Nong Y, Sun J, Wang Q, Zhai Z et al (2023) Triglyceride-glucose index is strongly associated with all-cause mortality in elderly females with diabetic foot ulcers: a 9-year follow-up study. Int Wound J. 10.1111/iwj.14344 37555254 10.1111/iwj.14344 PMC10777761

[24] Xu L, Wu M, Chen S, Yang Y, Wang Y, Wu S et al (2022) Triglyceride-glucose index associates with incident heart failure: a cohort study. Diabetes Metab 48(6):101365. 10.1016/j.diabet.2022.101365 35660526 10.1016/j.diabet.2022.101365

[25] Huang H, Li Q, Liu J, Qiao L, Chen S, Lai W et al (2022) Association between triglyceride glucose index and worsening heart failure in significant secondary mitral regurgitation following percutaneous coronary intervention. Cardiovasc Diabetol 21(1):260. 10.1186/s12933-022-01680-9 36443743 10.1186/s12933-022-01680-9 PMC9706938

[26] Zheng H, Chen G, Wu K, Wu W, Huang Z, Wang X et al (2023) Relationship between cumulative exposure to triglyceride-glucose index and heart failure: a prospective cohort study. Cardiovasc Diabetol 22(1):239. 10.1186/s12933-023-01967-5 37667253 10.1186/s12933-023-01967-5 PMC10476374

[27] Wang T, Xu J, Zhang H, Tao L, Huang X (2023) Triglyceride-glucose index for the detection of subclinical heart failure with preserved ejection fraction in patients with type 2 diabetes. Front Cardiovasc Med 10:1086978. 10.3389/fcvm.2023.1086978 36793475 10.3389/fcvm.2023.1086978 PMC9923050

[28] Chen Y, Fu J, Wang Y, Zhang Y, Shi M, Wang C et al (2023) Association between triglyceride glucose index and subclinical left ventricular systolic dysfunction in patients with type 2 diabetes. Lipids Health Dis 22(1):35. 10.1186/s12944-023-01796-1 36890516 10.1186/s12944-023-01796-1 PMC9993628

[29] Tuleta I, Frangogiannis NG (2021) Fibrosis of the diabetic heart: clinical significance, molecular mechanisms, and therapeutic opportunities. Adv Drug Deliv Rev 176:113904. 10.1016/j.addr.2021.113904 34331987 10.1016/j.addr.2021.113904 PMC8444077

[30] Nakamura M, Sadoshima J (2020) Cardiomyopathy in obesity, insulin resistance and diabetes. J Physiol 598(14):2977–2993. 10.1113/JP276747 30869158 10.1113/JP276747

[31] Wang X, Ni J, Guo R, Li L, Su J, He F et al (2022) SGLT2 inhibitors break the vicious circle between heart failure and insulin resistance: targeting energy metabolism. Heart Fail Rev 27(3):961–980. 10.1007/s10741-021-10096-8 33713009 10.1007/s10741-021-10096-8

[32] Pan KL, Hsu YC, Chang ST, Chung CM, Lin CL (2023) the role of cardiac fibrosis in diabetic cardiomyopathy: from pathophysiology to clinical diagnostic tools. Int J Mol Sci 24(10):864. 10.3390/ijms24108604 37239956 10.3390/ijms24108604 PMC10218088

[33] Sanz RL, Inserra F, Menendez SG, Mazzei L, Ferder L, Manucha W (2023) Metabolic syndrome and cardiac remodeling due to mitochondrial oxidative stress involving gliflozins and sirtuins. Curr Hypertens Rep 25(6):91–106. 10.1007/s11906-023-01240-w 37052810 10.1007/s11906-023-01240-w

[34] Palmiero G, Cesaro A, Vetrano E, Pafundi PC, Galiero R, Caturano A et al (2021) Impact of SGLT2 inhibitors on heart failure: from pathophysiology to clinical effects. Int J Mol Sci 22(11):5863. 10.3390/ijms22115863 34070765 10.3390/ijms22115863 PMC8199383

[35] Aroor AR, Mandavia CH, Sowers JR (2012) Insulin resistance and heart failure: molecular mechanisms. Heart Fail Clin 8(4):609–617. 10.1016/j.hfc.2012.06.005 22999243 10.1016/j.hfc.2012.06.005 PMC3457065

[36] Jaque-Fernandez F, Beaulant A, Berthier C, Monteiro L, Allard B, Casas M et al (2020) Preserved Ca(2+) handling and excitation-contraction coupling in muscle fibres from diet-induced obese mice. Diabetologia 63(11):2471–2481. 10.1007/s00125-020-05256-8 32840676 10.1007/s00125-020-05256-8

[37] Dia M, Gomez L, Thibault H, Tessier N, Leon C, Chouabe C et al (2020) Reduced reticulum-mitochondria Ca(2+) transfer is an early and reversible trigger of mitochondrial dysfunctions in diabetic cardiomyopathy. Basic Res Cardiol 115(6):74. 10.1007/s00395-020-00835-7 33258101 10.1007/s00395-020-00835-7 PMC7704523

[38] Merkely B, Hatala R, Wranicz JK, Duray G, Foldesi C, Som Z et al (2023) Upgrade of right ventricular pacing to cardiac resynchronisation therapy in heart failure: a randomised trial. Eur Heart J. 10.1093/eurheartj/ehad591 37632437 10.1093/eurheartj/ehad591 PMC10590127

