# Supplementary material for: Association between triglyceride-glucose index and the risk of heart failure hospitalization in older diabetic patients received right ventricular pacing: a retrospective cohort study
Source: Acta Diabetol. 2024 Jun 19;61(12):1527–36. doi: 10.1007/s00592-024-02322-0 (PMC11628445; doi:10.1007/s00592-024-02322-0)

**Figure S1.** Restricted cubic splines regression analysis of TyG index with HFH risk stratified by sex. Abbreviations: HFH, heart failure hospitalization; TyG index, triglyceride-glucose index.


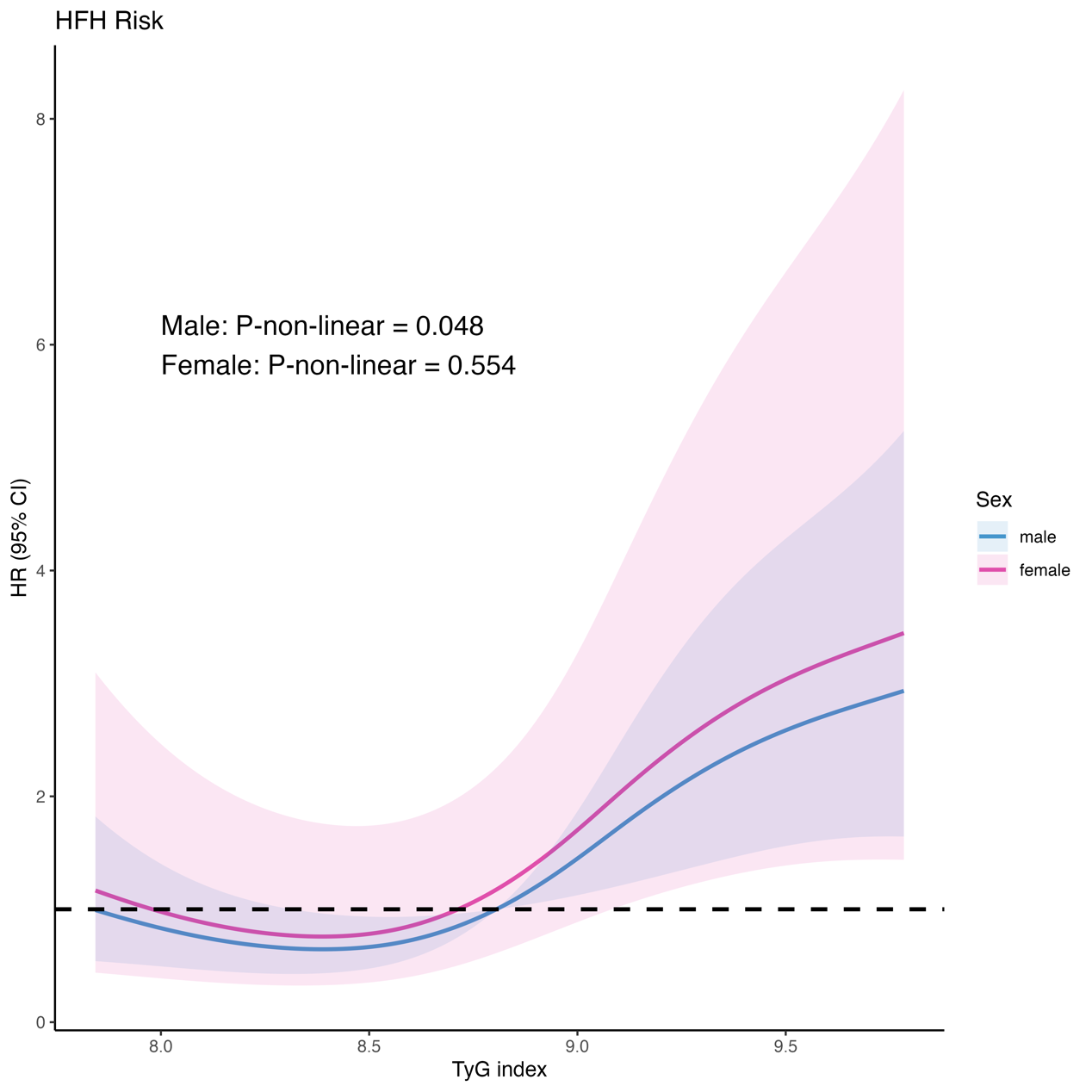


**Figure S2.** Restricted cubic splines regression analysis of TyG index with HFH risk stratified by AF. Abbreviations: HFH, heart failure hospitalization; TyG index, triglyceride-glucose index; AF, atrial fibrillation.


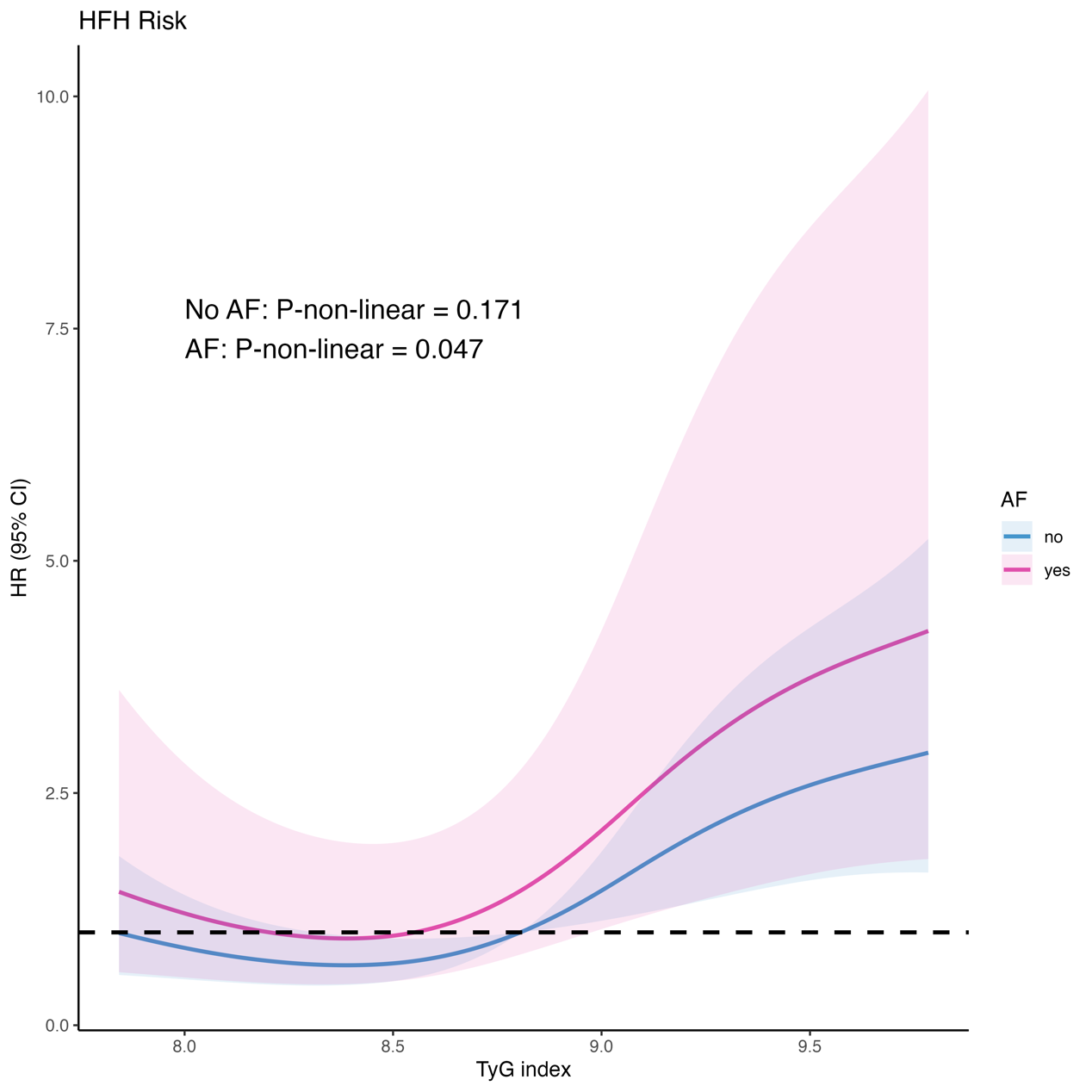


**Figure S3.** Restricted cubic splines regression analysis of TyG index with HFH risk stratified by CKD. Abbreviations: HFH, heart failure hospitalization; TyG index, triglyceride-glucose index; CKD, chronic kidney disease.

**
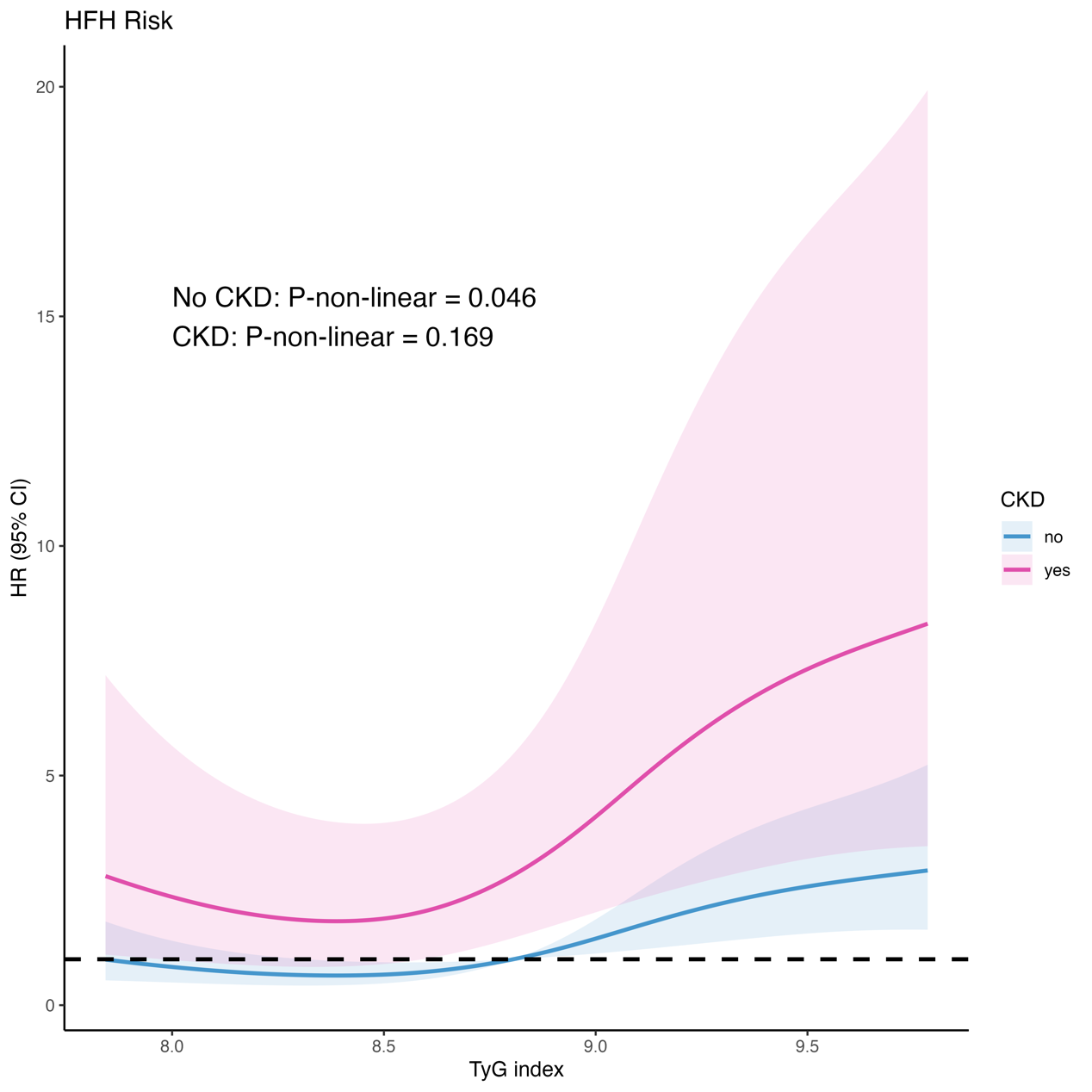
**

**Figure S4**. Subgroup and interaction analyses of the association between TyG index and HFH risk. Abbreviations: HFH, heart failure hospitalization; TyG index, triglyceride-glucose index; AF, atrial fibrillation; CKD, chronic kidney disease; PCI, percutaneous coronary intervention; CABG, coronary artery bypass grafting; LVEF, left ventricular ejection fraction; LVMI, left ventricular mass index.


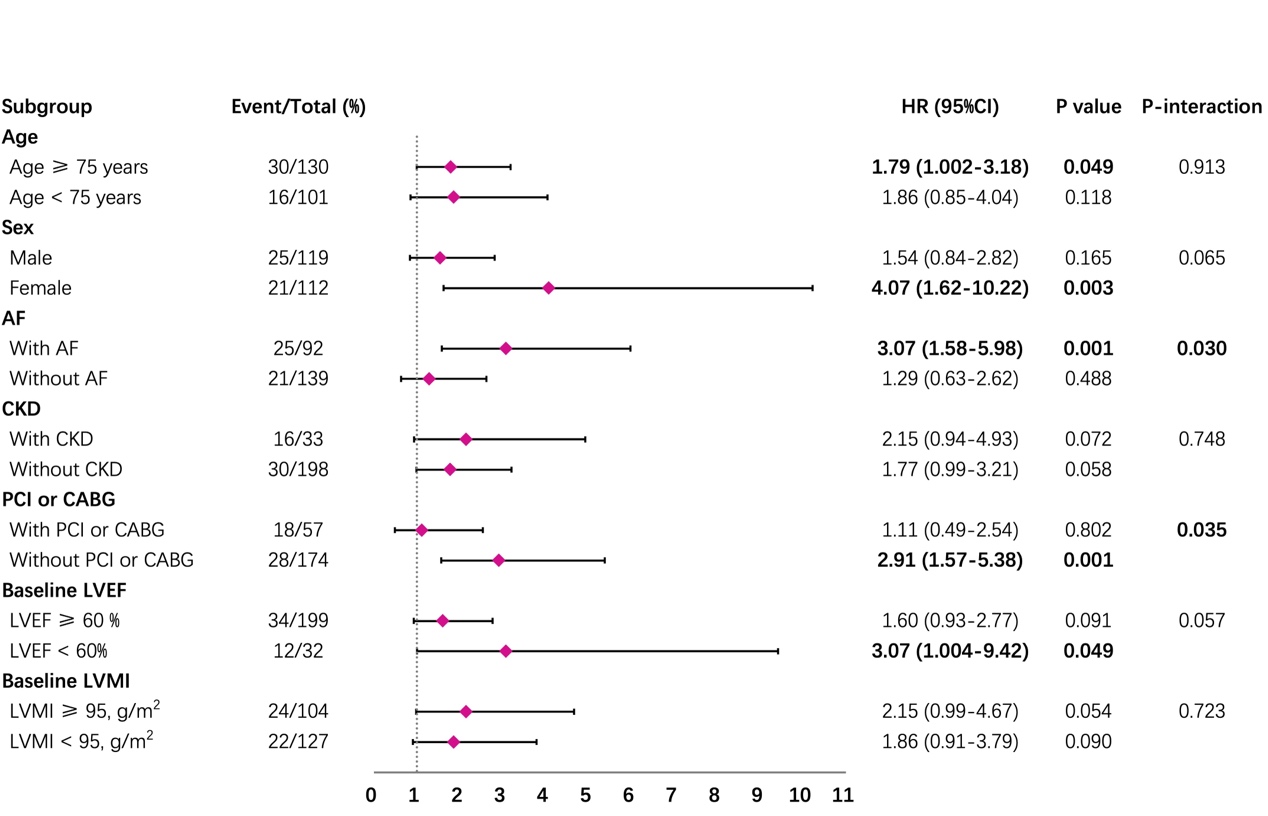

Supplement: Supplementary file 1 — Supplementary file1 (DOCX 14330 KB) [file 592_2024_2322_MOESM1_ESM.docx]
